# Croatian study on COVID-19-positive stroke patients during the second wave of the pandemic

**DOI:** 10.3325/cmj.2022.63.431

**Published:** 2022-10

**Authors:** Stela Rutović, Davor Sporiš, Miljenko Crnjaković, Gorana Vukorepa, Sabina Deveđija, Branko Malojčić

**Affiliations:** 1 Department of Neurology, Dubrava University Hospital, Zagreb, Croatia; 2 Department of Neurology, University Hospital Center Zagreb, Zagreb, Croatia

## Abstract

**Aim:**

To investigate stroke characteristics in patients with concomitant coronavirus disease 2019 (COVID-19) infection in Croatia during the second wave of the COVID-19 pandemic.

**Methods:**

This retrospective study investigated the characteristics of two groups of ischemic stroke patients: those who developed COVID-19 infection before stroke and those who developed the infection during the hospital stay after stroke onset. Stroke etiology was classified according to the Trial of Org 10172 in Acute Stroke Treatment (TOAST) classification.

**Results:**

We analyzed data from 255 stroke patients from 12 Croatian hospitals. The two groups of ischemic stroke patients differed in stroke etiology (*P* = 0.038). Patients with COVID-19 infection before stroke had fewer cardioembolic strokes (46% vs 29.1%), more cryptogenic strokes (32.5% vs 14.3%), and more strokes in multiple vascular territories (12.4% vs 1.8%). The percentage of large-vessel occlusions was high in both groups (49.6% and 44.4%). Median modified Rankin Scale score on discharge was 4 in both groups. Mortality was 36.4% in the group with stroke after COVID-19 and 33.3% in the group with COVID-19 after stroke.

**Conclusion:**

Ischemic stroke after COVID-19 differs in etiology from ischemic stroke complicated by COVID-19 infection. Both patient groups are characterized by severe disability and high mortality. Raising the awareness of prehospital stroke and optimization of clinical workflow are important if we want to improve the stroke outcomes by acute recanalization techniques.

Since the outbreak of coronavirus disease 2019 (COVID-19) pandemic, numerous neurological manifestations of the disease have been reported, including stroke and other cerebrovascular diseases (1). Several pathophysiological mechanisms of COVID-19-associated stroke have been proposed including coagulopathy, inflammation, endotheliopathy, platelet activation, and cardioembolism. COVID-19 patients more commonly have ischemic than hemorrhagic strokes. The former are characterized by many large-vessel occlusions, more frequent cryptogenic stroke etiology, and overall poor prognosis (2). The aim of this study was to investigate stroke characteristics in patients with concomitant COVID-19 infection in Croatia during the second wave of the COVID-19 pandemic. Due to specific mechanisms underlying stroke in the setting of COVID-19 infection, we hypothesized that types and frequencies of cerebrovascular events would differ between patients who developed COVID-19 before the onset of stroke and those who developed COVID-19 after hospital admission for stroke. We also hypothesized that these two groups of patients with ischemic stroke would significantly differ in stroke etiology.

## Methods

This retrospective cohort study was conducted between October 2020 and February 2021. The CROatian Study on COvid-19 Positive Stroke Patients during the Second Wave of the Pandemic (CROSCOPS2) study protocol, designed by researchers from Dubrava University Hospital, Zagreb, involved stroke patients from 12 Croatian hospitals (Supplementary Table 1(Supplementary material 1)). All patients were referred to Dubrava University Hospital for treatment. Data were extracted from the hospital database. The study was approved by the Ethics Committee of Dubrava University Hospital (2021/1312-07), which waived the informed consent.

The inclusion criteria were age older than 18 years, a radiologically confirmed diagnosis of ischemic or hemorrhagic stroke, subarachnoidal hemorrhage (SAH), cerebral venous sinus thrombosis, or transient ischemic attack (TIA), and a polymerase chain reaction (PCR)-confirmed COVID-19 infection. The exclusion criterion was a history of disability defined as modified Rankin score (mRS)>1. Participants were divided into two groups. The first group included patients who developed COVID-19 before stroke onset and who were hospitalized due to COVID-19 or stroke. The second group developed COVID-19 infection during hospital stay after stroke onset. Taking into account the median incubation period for COVID-19 of 5 days, the criteria for inclusion in the group with COVID-19 before stroke were positive reverse transcriptase (RT)-PCR test within 4 days of admission, even after the initial test was negative, because of low sensitivity of RT-PCR for SARS-CoV-2 in respiratory samples (3,4). This group also included patients who had clinical symptoms suggestive of COVID-19 infection at the time of admission and a positive test within 10 days of admission. The second group included patients with stroke and a hospital- acquired COVID-19 who were positive during hospital stay after stroke onset but did not satisfy the inclusion criteria for the first group.

The following data were compared between the two groups of patients with ischemic stroke:

1. Age and sex.

2. Stroke characteristics: stroke etiology based on the Trial of Org 10172 in Acute Stroke Treatment (TOAST) criteria (5); stroke severity assessed with the National Institute of Health Stroke Scale (NIHSS) score (6); presence of large vessel occlusion (LVO) (defined as occlusion of internal carotid artery, M1 and M2 segments of middle cerebral artery, A1 segment of anterior cerebral artery and basilar artery, and P1 segment of posterior cerebral artery); involvement of multiple vascular territories.

3. The interval between stroke onset and the occurrence of COVID-19 symptoms.

4. COVID-19 infection characteristics: presence of COVID-19 infection symptoms and/or radiologically verified pneumonia.

5. Comorbidities: hypertension, hyperlipidemia, atrial fibrillation, diabetes mellitus (DM) type 2, ischemic heart disease, congestive heart failure, carotid stenosis, body mass index (BMI)≥25, smoking, and previous stroke.

6. Laboratory findings on admission: white blood cell (WBC) count, neutrophil count, lymphocyte count, platelet count, C-reactive protein (CRP), alanine aminotransferase (ALT), aspartate aminotransferase (AST), lactate dehydrogenase (LDH), D-dimer, fibrinogen.

7. Treatment: treatment during hospital stay (intravenous thrombolysis [IVT], mechanical thrombectomy, mechanical ventilation, oxygen, corticosteroid, antibiotic, or heparin treatment), and medication before stroke onset (prior antiaggregant or anticoagulation therapy).

8. Outcome measures: neurological deterioration (defined as NIHSS increase of more than 4 during hospital stay that did not resolve until discharge, or recurrent stroke during hospital stay); hemorrhagic transformation (determined by ECASS II classification system); length of hospital stay; disability at discharge measured with mRS) (7); in- hospital mortality.

### Statistical analysis

Categorical data are presented as absolute and relative frequencies, and numerical data are presented as median, interquartile range, and total range. Differences between two independent sets of numerical data were assessed with a nonparametric Mann-Whitney U test, while differences between categorical data were assessed with a χ^2^ test and a Fisher exact test. Statistical significance level was set at 0.05. All *P* values were two tailed. Statistical analysis was performed with MedCalc, version 20.008 (MedCalc Software Ltd, Ostend, Belgium) or IBM SPSS Statistics, version 24.0 (IBM Corp., Armonk, NY, USA).

## Results

We collected data from 255 participants from 12 stroke centers (Supplementary Table 1). The median age was 74 years (67.3-82); 130 participants (50.9%) were men. Significantly more patients developed COVID-19 infection before stroke onset compared with patients who developed hospital-acquired COVID-19 infection after stroke onset (69.8% vs 30.2%, *P* < 0.001). A total of 214 patients (83.9%) had ischemic stroke, 24 patients (9.4%) had intracerebral hemorrhage (ICH), and a small number of patients developed other types of stroke and TIA. Baseline characteristics of all participants are presented in Table 1.

**Table 1:** Baseline characteristics (N = 255)

Characteristic	n (%)
Sex, male	130 (50.9)
Age in years, median (interquartile range)	74 (67.3-82)
Stroke after COVID-19*	178 (69.8)
COVID-19 after stroke*	77 (30.2)
Types of cerebrovascular events	
ischemic stroke	214 (83.9)
intracerebral hemorrhage	24 (9.4)
subarachnoidal hemorrhage	5 (2.0)
cerebral venous sinus thrombosis	4 (1.6)
transient ischemic attack	8 (3.1)

*P < 0.001.

Patients who developed COVID-19 before stroke and patients who developed COVID-19 after stroke significantly differed in the type and frequency of cerebrovascular events (*P* = 0.021). The number of patients with ischemic stroke was similar in both groups, while the number of patients with intracerebral hemorrhage was two times higher in the group with COVID-19 after stroke. All patients with SAH and TIA were in the first group (Table 2).

**Table 2:** Types and frequencies of cerebrovascular events in patients who developed stroke after coronavirus disease 2019 (COVID-19) and in patients who developed COVID-19 after stroke

Types of cerebrovascular events	No. (%) of patients with		
	stroke after COVID-19 (N = 178)	COVID-19 after stroke onset (N = 77)	P
Ischemic stroke	151 (84.8)	63 (81.8)	0.021
Intracerebral hemorrhage	12 (6.7)	12 (15.6)	
Subarachnoidal hemorrhage	5 (2.8)	0	
Cerebral venous sinus thrombosis	2 (1.1)	2 (2.6)	
Transient ischemic attack	8 (4.5)	0	

In patients with ischemic stroke, the two groups did not significantly differ in age, sex, or stroke severity categorized according to NIHSS score. There was a significant difference in stroke etiology assessed with the TOAST classification (*P* = 0.038). In the group with COVID-19 after stroke, there were more cardioembolic strokes than in the group with stroke after COVID-19 (46% vs 29.1%). In the group with stroke after COVID-19, there were more cryptogenic strokes (32.5% vs 14.3%). The groups did not significantly differ in the number of strokes with LVO, while the number of strokes in multiple vascular territories was significantly higher in the group with stroke after COVID-19 (*P* = 0.023). The number of patients with symptomatic COVID-19 infection was higher in the group with stroke after COVID-19 (*P* = 0.007), while the median number of days between the onset of COVID-19 symptoms and stroke onset was higher in the group with COVID-19 after stroke (*P* < 0.001). The number of patients who developed pneumonia did not differ between the groups. There was no significant difference in the distribution of comorbidities, except for atrial fibrillation, which was more frequent in the group with COVID-19 after stroke (*P* = 0.018).

The group with stroke after COVID-19 had lower lymphocyte count and higher CRP, LDH, D-dimer, and fibrinogen. There was no difference in the number of patients who were treated with IVT or mechanical thrombectomy or were mechanically ventilated. In the group with stroke after COVID-19, more patients received corticosteroid treatment (*P* = 0.047). There was no significant difference in the number of patients who received antibiotic treatment, oxygen, or heparin therapy. Significantly more patients with COVID-19 after stroke received prior antiaggregant (*P* = 0.008) and anticoagulant therapy (*P* = 0.031). The length of hospital stay was higher in the group with COVID-19 after stroke (*P* < 0.001). The number of patients with neurological deterioration was higher in the group with stroke after COVID-19, although the difference did not reach significance (*P* = 0.054). There was no significant difference in other measures of outcome, including hemorrhagic transformation, median mRS on discharge (4 in both groups), and in-hospital mortality (36.4% in the group with stroke after COVID-19 and 33.3% in the group with COVID-19 after stroke). Comparisons for all patients with ischemic stroke are presented in Table 3.

**Table 3:** Comparison of characteristics of ischemic stroke patients who developed stroke after coronavirus disease 2019 (COVID-19) and those who developed COVID-19 after stroke onset

	No. (%) of patients with		
Parameters	stroke after COVID-19	COVID-19 after stroke onset	P
**All strokes**	151	63	
**Sex, male**	70 (46.4)	33 (52.4)	0.423
**Age, years, median (IQR)**	76 (69-82)	73 (63.5-80.8)	0.082
**NIHSS, median (IQR)**	8 (4-15)	7 (4.3-12.8)	0.282
**TOAST**	151	63	
large artery atherosclerosis	33 (21.9)	13 (20.6)	0.038
cardioembolism	44 (29.1)	29 (46.0)	
small vessel	18 (11.9)	10 (15.9)	
other determined	7 (4.6)	2 (3.2)	
undetermined	49 (32.5)	9 (14.3)	
**LVO**	60 (49.6)	24 (44.4)	0.531
**Multiple vascular territories**	18 (12.4)	1 (1.8)	0.023
**Symptomatic COVID-19 infection**	112 (74.2)	35 (55.6)	0.007
**COVID-19 to stroke interval in days, median (IQR)**	5 (0 to 9)	-8.5 (-17 to -6)	<0.001
**Pneumonia**	97 (64.2)	36 (57.1)	0.334
**Comorbidities**			
hypertension	129 (86.6)	52 (83.9)	0.609
hyperlipidemia	73 (48.3)	33 (52.4)	0.586
atrial fibrillation	47 (31.1)	30 (47.6)	0.018
diabetes mellitus type 2	49 (32.5)	23 (36.5)	0.573
previous stroke	33 (21.9)	21 (33.3)	0.081
ischemic heart disease	35 (23.2)	13 (20.6)	0.686
congestive heart failure	27 (17.9)	12 (19.0)	0.842
carotid stenosis	18 (11.9)	12 (19.0)	0.172
smoking	13 (10.5)	9 (16.1)	0.291
body mass index ≥25	18 (14.1)	7 (12.1)	0.714
**Laboratory findings**			
white blood cell count ×10^9^/L, median (IQR)	9.1 (6.4-12.1)	9.1 (6.7-12.2)	0.839
neutrophil count ×10^9^/L, median (IQR)	6.4 (4.4-8.8)	6.0 (4.2-9.1)	0.971
lymphocyte count ×10^9^/L, median (IQR)	1.0 (0.6-1.5)	1.7 (1.2-2.2)	<0.001
platelet count ×10^9^/L, median (IQR)	224 (170.5-294.5)	232 (195.5-321.5)	0.173
CRP mg/L, median (IQR)	44.9 (10.7-104.9)	11.1 (4.5-41.9)	0.001
ALT U/L, median (IQR)	25 (17-41)	24 (14.3-39.3)	0.512
AST U/L, median (IQR)	29 (20-44)	26.5 (22-47)	0.636
LDH U/L, median (IQR)	297 (208-415)	238 (191.8-262.8)	0.004
D-dimer μg/L, median (IQR)	2.1 (0.8-4.4)	0.9 (0.5-2.4)	0.007
fibrinogen g/L, median (IQR)	5.4 (4.0-6.4)	4.0 (3.6-4.9)	0.004
**Treatment**			
IVT	7 (4.6)	5 (7.9)	0.343
mechanical thrombectomy	7 (4.6)	5 (7.9)	0.343
mechanical ventilation	18 (11.9)	4 (6.3)	0.221
oxygen therapy	89 (58.9)	33 (53.2)	0.441
corticosteroid treatment	79 (53.0)	24 (38.1)	0.047
antibiotic treatment	96 (64.4)	47 (74.6)	0.154
heparin treatment	128 (85.9)	56 (88.9)	0.557
prior antiaggregation therapy	44 (29.5)	29 (49.2)	0.008
prior anticoagulation therapy	26 (17.4)	19 (30.6)	0.031
**Outcome**			
deterioration	24 (16.2)	4 (6.3)	0.054
hemorrhagic transformation	11 (11.1)	6 (13.0)	0.742
length of hospital stay in days, median (IQR)	11 (6-20)	22 (12-33)	<0.001
mRS on discharge, median (IQR)	4 (2-6)	4 (2-6)	0.941
in-hospital mortality	55 (36.4)	21 (33.3)	0.673

*Abbreviations: NIHSS – National Institute of Health Stroke Scale; TOAST – Trial of Org 10172 in Acute Stroke Treatment; LVO – large vessel occlusion; CRP – C-reactive protein; ALT – alanine aminotransferase; AST – aspartate aminotransferase; LDH – lactate dehydrogenase; IVT – intravenous thrombolysis; mRS – modified Rankin score.

## Discussion

In this study, the two patient groups significantly differed in the types and frequency of cerebrovascular events. Most of the patients in both groups had an ischemic stroke, while the number of hemorrhagic strokes was higher in the group with COVID-19 after stroke. According to a meta- analysis by Nannoni et al (1), acute cerebrovascular disease occurred in 1.4% of patients with COVID-19, with a clear predominance of acute ischemic stroke over intracerebral hemorrhage (87.4% vs 11.6%).

Most of the previous studies compared stroke patients with COVID-19 to contemporary or historical controls without COVID-19 (8-11). To our knowledge, this is the first study comparing patients with ischemic stroke who developed COVID-19 infection before the stroke onset with patients who developed COVID-19 infection after the stroke onset. The UK- multicenter case-control study by Perry et al (8) compared stroke patients with COVID-19 with contemporary controls without COVID-19. The study used the same criteria as we did to define COVID-19-positive stroke patients, but they included all patients with ischemic and hemorrhagic stroke, not specifically analyzing ischemic stroke characteristics.

In this study, the two groups did not differ in age, sex, or the NIHSS score, the latter being moderately high in both groups. Several studies comparing stroke patients with and without COVID-19 found no significant difference in age and gender (8,9), while others found that ischemic strokes caused by COVID-19 were more likely to occur in men and in younger patients (10,11). Furthermore, strokes in patients with COVID-19 resulted in higher NIHSS score, which might be a consequence of direct vasculopathic effects of SARS-CoV-2, as well as of a higher number of LVO (8,12,13). Furthermore, a reduced number of patients with milder strokes were admitted to hospital during the pandemic, probably due to the fear of in-hospital infection or social distancing measures (12).

We found LVO in 49.6% of patients with stroke after COVID-19 and in 44.4% of patients with COVID-19 after stroke. Similar rates have been reported elsewhere (11,13). High number of LVO in our group with COVID-19 after stroke can be explained by many cardioembolic strokes (46%) in this group.

The number of patients with cryptogenic stroke was two times higher in the group with stroke after COVID-19. A meta-analysis by Luo et al showed an increased prevalence of cryptogenic strokes in patients with stroke and COVID-19, with a pooled proportion of 35% (10). This may be a consequence of an unknown mechanism of stroke caused by COVID-19, but also of the unavailability of extended diagnostic assessment for all stroke patients initially diagnosed with COVID-19 due to organizational changes at hospital wards (14).

We found a similar number of patients with small-vessel strokes in both groups. Some studies did not find any differences in the prevalence of small-vessel infarctions between COVID-19- related stroke and non-COVID-19 stroke (8,12). Other studies showed that stroke in patients with COVID-19 was less frequently caused by small-vessel occlusion, which may be a consequence of different mechanisms of stroke in the setting of COVID-19 infection. Furthermore, strokes caused by small-vessel disease usually have mild to moderate symptoms, and such patients were less likely to present at medical centers during the COVID-19 pandemic (11,15).

In our study, the number of strokes in multiple vascular territories was significantly higher in the group with stroke after COVID-19. This finding is in line with previous results (1,15) and may be a consequence of coagulopathy and thromboembolic events. The groups did not differ in the rates of IVT and mechanical thrombectomy, which were low in both groups. The current studies indicate a negative impact of COVID-19 on the utilization of acute recanalization techniques in stroke patients, showing a decline in the number of patients who receive IVT and mechanical thrombectomy (16).

Most of the patients in both groups had pre-existing vascular risk factors, with hypertension being the most prevalent in both groups. There was no significant difference in comorbidities, except atrial fibrillation, which was more common in the group with COVID-19 after stroke. We assume that the patients with stroke caused by atrial fibrillation had more severe neurological deficit, which increased the risk of developing COVID-19.

Earlier studies also reported that the majority of patients with ischemic stroke associated with COVID-19 had common vascular risk factors. Qureshi et al (9) did not find any difference in comorbidities between stroke patients with and without COVID-19. Patients with COVID-19 and stroke were less likely to have hypertension and a history of stroke or TIA (13), as well as a history of smoking or atrial fibrillation than patients without COVID-19 (17).

We found lower lymphocyte count, higher C-reactive protein, higher D-dimer, fibrinogen, and LDH levels in patients who had stroke after COVID-19. Similar findings have been reported in other studies on COVID-19 stroke patients (13,15,18).

Mortality and disability were high in both groups. High mortality rates and severe disability have been reported in previous studies (1,8,12,18). Higher mortality rates observed by Yaghi et al (13) may be explained by their study including more patients requiring mechanical ventilation and having a higher NIHSS score on admission than it was the case in our study. This difference in stroke severity can, as mentioned earlier, be a consequence of the observed trend for patients with milder strokes to stay at home. This trend was probably less pronounced in our patients, possibly because in our case COVID-19- positive patients were treated in a specialized respiratory center, which may have decreased the fear of acquiring nosocomial infection in patients with minor stroke.

Worse outcomes in both groups of our patients may be potentiated by multiple complications of COVID-19, such as cytokine release syndrome, acute respiratory distress syndrome, multiple organ failure, myocardial injury, acute coronary syndrome, cardiac arrhythmias, coagulopathy, and pulmonary embolism (19).

Old age, severe stroke, cortical infarction, and comorbidities increase the risk of nosocomial infections in stroke patients (20). We assume that these factors also increase the risk of acquiring in-hospital COVID-19 infection. Atrial fibrillation may be the most important predictive factor of LVO, resulting in severe stroke or cortical stroke.

Hospital-acquired COVID-19 infection after stroke onset significantly prolonged the hospital stay in patients in the group with COVID-19 after stroke, which was two times longer than in the group with stroke after COVID-19. This can lead to a higher socioeconomic burden to the hospital sector.

A strength of this study is that it is the first national study on COVID-19-related stroke. Its major limitation is the retrospective observational design, so some data were not available for all patients. Possibly not all COVID-19 symptomatic patients were recognized, which may have caused some patients to be included in the wrong group.

In conclusion, ischemic stroke after COVID-19 differs in etiology from ischemic stroke complicated by COVID-19 infection. Ischemic stroke patients who acquire COVID-19 in hospital are more likely to have atrial fibrillation and higher NIHSS score. Both patient groups had high rates of LVO, severe disability, and high mortality rates. Raising prehospital stroke awareness and optimization of clinical workflow are important if we want to improve the stroke outcomes by acute recanalization techniques.
