# Supplementary material for: Croatian study on COVID-19-positive stroke patients during the second wave of the pandemic
Source: Croat Med J. 2022 Oct;63(5):431–7. doi: 10.3325/cmj.2022.63.431 (PMC9648083; doi:10.3325/cmj.2022.63.431)
Supplement: Supplementary Table 1 [file CroatMedJ_63_s005.pdf]

Supplementary Table 1. Number of patients referred from each institution to Dubrava University Hospital

Dubrava University Hospital, Zagreb	34
Zagreb University Hospital Center	58
Sestre Milosrdnice University Hospital Center, Zagreb	29
Sveti Duh University Hospital, Zagreb	13
University Hospital Center Osijek	31
University Hospital Center Rijeka	21
University Hospital Center Split	8
General Hospital Varaždin	28
General Hospital Slavonski Brod	16
General Hospital Zadar	7
County Hospital Čakovec	5
General County Hospital Vinkovci	5
